# mHealth Support to Stimulate Physical Activity in Individuals With Intellectual Disability: Protocol for a Mixed Methods Pilot Study

**DOI:** 10.2196/37849

**Published:** 2022-09-15

**Authors:** Henriette Michalsen, Silje C Wangberg, Gunnar Hartvigsen, André Henriksen, Gunn Pettersen, Letizia Jaccheri, Reidun Birgitta Jahnsen, Gyrd Thrane, Cathrine Arntzen, Audny Anke

**Affiliations:** 1 Department of Rehabilitation University Hospital of North Norway Tromsø Norway; 2 Department of Health and Care Sciences Faculty of Health Sciences UiT – The Arctic University of Norway Narvik Norway; 3 Department of Computer Science Faculty of Science and Technology UiT – The Artic University of Norway Tromsø Norway; 4 Department of Health and Care Sciences Faculty of Health Sciences UiT – The Arctic University of Norway Tromsø Norway; 5 Department of Computer Science Faculty of Information Technology and Electrical Engineering NTNU Trondheim Norway; 6 Institute of Health and Society, Research Centre for Habilitation and Rehabilitation Models and Services (CHARM) Faculty of Medicine University of Oslo Oslo Norway; 7 Department of Clinical Medicine Faculty of Health Sciences UIT - The Arctic University of Norway Tromsø Norway

**Keywords:** intellectual disability, physical activity, technology, mHealth, mobile health, exercise, protocol, technology, pilot study, trial, caregivers

## Abstract

**Background:**

Several studies have shown that individuals with intellectual disabilities (IDs) have low levels of physical activity (PA), and intervention studies on PA suggest inconsistent evidence. The use of technology as a means of motivation for PA has yet to be extensively explored and needs to be further investigated.

**Objective:**

We aim to assess the feasibility and acceptability of procedures for an intervention arm in a future trial on mobile health (mHealth) to support PA for individuals with IDs. In addition, we aim to examine how the use of technology can influence motivation for PA among participants, their caregivers, and staff members.

**Methods:**

A mixed methods pilot study of an intervention arm will be carried out in a planned randomized controlled trial (RCT). Ten participants with ID and their caregivers or a staff member will be included. Information will always be provided by a caregiver or a staff member, or participants with ID if possible. Assessments will be carried out at baseline, follow-up after 4 weeks, and 12 weeks, and include questionnaires on PA, social support, self-efficacy, and challenging behavior. PA will be measured with 2 different activity trackers (Fitbit and Axivity) for 1 week at all assessments. Feasibility will be assessed as recruitment and adherence rate, missing data, usability of the motivational mHealth tool, and estimates of effectiveness. Acceptability of study procedures, activity measures, and motivation for participation in PA will be additionally assessed with qualitative methods at the end of the intervention.

**Results:**

Enrollment commenced in May 2021. Data collection was completed in March 2022.

**Conclusions:**

This pilot study will evaluate the feasibility and acceptability of study procedures of the intervention arm of a planned RCT to address feasibility issues, improve study procedures, and estimate effectiveness of the study measures. How the use of technology can influence motivation for PA will also be examined, which can help guide and improve future PA interventions involving the use of technology.

**Trial Registration:**

ClinicalTrials.gov NCT04929106; https://clinicaltrials.gov/ct2/show/NCT04929106

**International Registered Report Identifier (IRRID):**

DERR1-10.2196/37849

## Introduction

### Physical Activity and Intellectual Disability

Compared to the general population, individuals with intellectual disability (ID) have more sedentary lifestyles, poor health, and greater barriers for participation in physical activity (PA), and consequently lower levels of PA [1-6]. PA is beneficial for cardiovascular and muscular capacity and psychological well-being and can reduce the burden of chronic disease for individuals with ID [1,7-9]. A systematic review by Hassan et al [10] investigated the efficacy of randomized controlled PA interventions, and the results showed inconsistent evidence, with only 3 intervention studies showing a prolonged effect of the intervention [8,10-12]. It is clear that new approaches and further investigations are needed to develop effective interventions for PA in this group.

Including individuals with ID in clinical intervention trials can be challenging, since there are several barriers in all aspects of the research process [13,14]. Struggling to inform the individual with ID about the research, difficulties in obtaining informed consent, finding transport to research sites, and following instructions are common challenges in clinical trials involving individuals with ID [3,15,16]. It is, therefore, particularly important to carefully plan intervention studies for this group, and performing pilot trials or feasibility studies can improve the quality of research [17].

### Use of Technology for Individuals With ID

Recent studies have shown measurable benefits from the use of mobile technologies in health care and the use of technology in everyday life has been explored and deemed promising for individuals with ID [18-21]. Although some studies have explored the possibility of using mobile technologies for promoting PA for individuals with ID, there has been only one preliminary report of a randomized controlled trial (RCT) using smartphone support to increase motivation for PA in youths and adults with ID [22-25]. In Norway, many individuals with ID have a smartphone or a tablet device they can use for tailored PA interventions and this use has not been tested. A previous study in this research project showed that individuals with ID are motivated to participate in PA and evince an interest in technology [26,27]. According to the *World Report on Disability 2011* [28], health promotion efforts targeting this population can improve lifestyle behaviors. The report states that these individuals have the right to be included in all PA programs.

### Objectives

The primary objective of this pilot study is to assess feasibility and acceptability of study procedures for a future RCT. We also aim to explore the experience of participants and their caregivers regarding the intervention, focusing on success factors, problems, and reasons for dropout, and to understand how the use of technology can influence motivation for PA for this group.

## Methods

### Study Context

This pilot study is part of a larger project, which aims to develop and test tailored mobile health (mHealth) support systems that promote PA in individuals with ID. We have previously conducted a qualitative study on motivation for participation in PA for such individuals, held workshops and collaborated with developers, and performed usability tests [26,29]. The results from this study will be used in the planning of a larger RCT with 60 participants. See Michalsen et al [27] for more detailed information regarding the RCT.

### Design

This protocol describes a mixed methods pilot study of an intervention arm in a planned RCT [27].

### Participant Selection and Recruitment

Eligible participants will be identified through their participation in a larger study on health indicators for individuals with ID, the Norwegian Health in Intellectual Disability (NOHID) study, and through staff leaders at municipality levels who have identified potential participants [30]. Overall, 10 participants with ID as well as caregivers and support persons (ie, staff) will be included if they have a sedentary lifestyle or low levels of PA. A sedentary lifestyle is defined as primarily engaging in reading, watching TV, or other mainly sedentary activities. A low level of PA is defined as mainly walking or other light PA for less than 4 hours a week. The number of participants is chosen on the basis of assumptions about sufficient variation for the investigation of feasibility and acceptability of the pilot study [23]. Data saturation of qualitative data collected will be evaluated continually and there will be scope for flexibility to achieve this during the data collection period.

### Inclusion and Exclusion Criteria

The inclusion criteria are as follows: (1) low levels of PA, (2) diagnosis of ID (mild, moderate, severe, or profound), (3) aged between 16 and 60 years, (4) ability to participate in the pilot study, (5) capable of walking with or without support, and (6) can provide written informed consent or consent can be obtained from a legal representative. Prior to enrollment, all participants will be screened for readiness, and, if necessary, medical clearance will be obtained. The Physical Activity Readiness Questionnaire will be used for this purpose [31]. Exclusion criteria are as follows: (1) medical contraindications for participation in programs with increased exercise, as advised by the primary care or ID specialist physician; (2) high levels of PA; and (3) inability to provide written informed consent or consent cannot be obtained from a legal representative.

### Ethics Approval

All participants and their caregivers will receive information about the purpose and procedures for the study. Informed consent will be obtained from the individuals with ID, as far as possible, if the person has decision-making capability to provide consent. In addition, or in case the person with ID cannot provide consent, a caregiver will provide informed consent on behalf of the person with ID. The definite trial and the pilot study have been approved by the Regional Committees for Medical and Health Research Ethics in Norway (2016/1770) and the data protection officer at the University Hospital of North Norway.

### Intervention

The final app is named Active Leisure (Aktiv Fritid) and consists of an advanced activity planner based on the platform developed by the nonprofit organization Smart Cognition AS (Smart Cognition AS). The app offers various interface options (symbols only, easy-to-read text, plain text, and read aloud). See Multimedia Appendix 1 for interface options for the Active Leisure app. The activity planner will mostly be used by an individual with ID and a support person (caregiver or health care provider). Participants will have different levels of functioning and will require different proximity of support persons, which will be considered when delivering the intervention. After completing an activity, a simple reward is available (eg, a smiling face or to share a picture with other users of the app). All activities that go into the activity planner are added through a web application. For this project, the principal author (HM) will be responsible for adding personalized activities into the activity planner for each participant. The use of tailored communication [32] through personalization (the use of the individual’s first name in the activity planner), adaptation (individually chosen activities, adjusted communication, including symbols, easy-to-read text, or plain text), preparation and planning, and feedback (rewarding and positive feedback after activities have been performed) are expected to increase motivation and thereby lead to higher levels of PA.

An individual mHealth exercise app has also been developed as a potential alternative that can be added to the Active Leisure planner; this app is called Sorterius (see Multimedia Appendix 2) [33] and is an augmented reality game, inspired by the popular game Pokémon Go. In this app, individuals can use the camera on their phone to look for virtual waste on the ground. Virtual waste will appear as they walk and can be sorted into correct waste bins, depending on the level of difficulty chosen (easy=1 waste bin, medium=2 waste bins, difficult=4 waste bins). When a set number of items has been collected, the individual will receive a virtual reward (stars and positive feedback). It is possible to add goals for steps per day and a weekly goal, which can be tailored to each individual’s interest.

### Data Collection

#### Procedure

Three research nurses from the clinical trial unit at the University Hospital of North Norway will support study procedures. Participants will be screened for readiness via telephone. The participants’ closest relative or guardian or a support person approved by the participants will be contacted for a baseline assessment via telephone. Questionnaires will be sent over email using the electronic system Research Electronic Data Capture (REDCap), a web-based system that is compliant with relevant regulations and security requirements. The study coordinator will evaluate the data of all participants for completeness. In cases of missing data, respondents will be contacted. Two activity trackers (Fitbit and Axivity), to be worn for 7 consecutive days, will be handed to participants after the baseline assessment. According to the instructions, Fitbit will be used on the dominant hand and Axivity on the nondominant hand. Participants who only accept to use one of the activity trackers will use the Fitbit device, as PA output from this device will be used to assess study outcomes in a later definite trial. After baseline assessments, participants and caregivers or staff will be invited to a goal-setting meeting where the Goal Attainment Scale will be used, and they will be introduced to the mHealth application Active Leisure and a motivating augmented reality application for sorting waste, called “Sorterius” [33]. Caregivers or staff will receive a telephone call from the study nurse at 4-week follow-up and asked to complete the questionnaires via email. The study nurse will also as ask caregivers or staff about the use of the activity trackers and ask for general comments regarding study procedures. These data will be added in the electronic system REDCap. Subsequently, they will receive the activity trackers again. The same procedure will be followed at the 12-week follow-up. Technical support will be available during the study period. After the end of the pilot trial, all participants and caregivers or support persons or staff will be asked to take part in a semistructured interview to share their experiences from the study.

#### Demographics, Level of ID, and Baseline Functioning

Background data of the participants, such as age, gender, educational level, marital status, living condition, employment status, educational status, job-related or day-center activities, leisure time activities, smoking habits, level of ID (ie, mild, moderate, severe, or profound), genetic diagnosis, medical history or readiness for the PA intervention, and use of medication will be collected at baseline. Ratings of the 5-level Gross Motor Function Classification System and the Communication Function Classification System will also be collected during baseline assessments [34-36].

#### Qualitative Interview

The recruited participants and their caregivers or a staff member in the pilot study will be asked to participate in a 1-hour qualitative interview after the 12-week follow-up assessment. A semistructured interview will take place at the participant’s home or other locations convenient for the participant, caregiver, or staff member [37]. Given the cognitive and speech limitations of many people with ID, semistructured interviews will be performed with the person with ID along with a caregiver or staff member, or only performed with the caregiver or staff member. Therefore, at least for the proxy interviews comprising participants unable to answer the questions, the interviews need to be arranged to facilitate an interpretation that reflects the participants opinions or attitudes. An interview guide has been developed and will be used in the interviews [26]. The interview guide will be categorized into 2 sections. Section 1 will focus on feasibility and acceptability of procedures, how the mHealth support will be used, and participant and caregiver or staff experiences of participation in all aspects of the study. Section 2 will focus on motivation for using technology to enhance PA levels, how the environment around the person with ID can use technology for PA, and practical barriers for using technology in an everyday setting. All interviews will be audiotaped, transcribed verbatim, and anonymized.

#### Aspects of Feasibility

To assess feasibility, recruitment, adherence to the intervention, retention, and completeness of data will be evaluated. Recruitment will be evaluated from the number of invited participants, eligible, and recruited or not recruited participants. We expect to include 1-2 eligible participants with ID per week. The expected recruitment rate is 20%; that is, 20% of eligible individuals are expected to agree to be enrolled. Adherence to the intervention will be evaluated through information about the actual use of the applications, which will be obtained from the qualitative interviews. Retention is defined as the number of participants who complete all 3 assessments and the qualitative interview. We expect 80% of the participants to complete the assessments and the interviews. Completeness of quantitative data (missing) will be recorded, and less than 10% data are expected to be missing. Adverse events will be reported to the researchers or the study nurses.

#### Outcome Measures in the Definite RCT

To assess the planned primary outcome in the definite trial PA level (accelerometer measured steps per day) [26], the participants will use one Fitbit device (Versa). The watch will show a neutral screen during baseline and follow-up assessments. Participants who consent will, in addition, use one device manufactured for scientific purposes (Axivity AX3). Axivity will be used to assess the validity of the Fitbit Versa. This will objectively assess PA and sedentary time [38]. The PA level will be measured for 7 days at each assessment, with a minimum of 3 consecutive days of measurement [8]. Only days with at least 10 hours of wear time will be considered valid [39]. Many of the commercial fitness trackers have been validated for use in the research, including devices from Fitbit [40-42]. The primary outcome in the definite trial will be objectively measured PA assessed by increase in steps per day from the Fitbit device for each participant.

Several secondary outcome measures are explored: minutes of moderate activity, the International Physical Activity Questionnaire Short, BMI, and goal attainment [43-46]. Proxy respondent measures include measures of aberrant behavior (the Aberrant Behaviour Checklist-Community), social support for PA and self-efficacy in a PA setting, and health status (the EQ-5D scale) [47-49]. To control for adverse effects, an adapted quality-of-life scale will be assessed [50]. These measures will be used as evaluation of the sensitivity of the data to detect change and as part of testing the procedures for a later RCT. All these measures have previously been used in research with individuals with ID, and more information about each of these measures can be found in the protocol article for the planned RCT [27].

### Data Analyses

#### Quantitative Data

Appropriate quantitative statistical analyses will be performed using SPSS (version 28; IBM Corp) in accordance with the type and distribution of data. The descriptive statistics are presented as median (IQR) values, means with SDs or 95% CIs, and frequencies of categorical data. The distributional properties of variables will be examined. Hypothesis testing will not be performed. In concordance with the Consolidated Standards of Reporting Trials (CONSORT) 2010 extension, estimates of effects using participant outcome measures (differences from baseline to postintervention) will be explored and reported as estimates with 95% CIs without *P* values [51,52]. We shall also perform mixed analyses; quantitative data can be supplemented from information in the qualitative material. For instance, if there is a change in the activity data, interviews can shed light on whether changes are related to the use of technology or whether other mechanisms are involved.

#### Qualitative Data

The transcribed interviews will be analyzed in accordance with the principles of systematic text condensation [53]. This analysis follows a 4-step procedure. One author will perform the analysis and coding, while the other authors will contribute to further analysis, discussing interview content and preliminary findings, themes, and subthemes. The qualitative approach delivers data on feasibility of procedures, expectations, and the use of applications for PA, experiences with activity trackers and motivation for using technology to increase PA. In addition, clarity and discussion of minor themes and diverse cases will also be included to enhance trustworthiness and transferability to the findings. Reflexivity is a key issue to reflect on the conditions that might corrupt transferability and to adequately interpret the findings [53]. This reflection is imperative, particularly since the authors in this project strongly believe in the need for PA among individuals with ID as well as in the effect of the intervention approach. To facilitate such reflection, the coauthor validation procedure will be important. The consolidated criteria for reporting qualitative research will be utilized to ensure highest possible quality research in this pilot study [54].

## Results

Enrollment was initiated in May 2021 and was completed in March 2022. The participants were recruited and administered the intervention individually.

The main contribution of this protocol is a detailed description of a pilot trial that will produce knowledge on how to test procedures of future RCTs for PA among individuals with ID. In addition, this pilot trial will investigate how such individuals can be motivated to use mHealth tools. The trial is registered in ClinicalTrials.gov (NCT04929106).

## Discussion

### Expected Findings

In this pilot study, a possible main finding will be the large variations in the quality of the collected data. Many intervention studies including individuals with ID have recruitment problems [13], report that data are missing [12], and have a low participation rate, particularly for individuals with severe ID [5,6]. Conducting pilot trials is, therefore, important when planning RCTs since it offers the possibility to address feasibility issues, improve procedures, and prevent wastage of resources in future trials [52]. This knowledge can guide future development of technology-based PA programs and help improve future intervention studies aiming to improve levels of PA.

We expect that this pilot trial will uncover possible challenges in using technological devices for interventions aiming at PA promotion for individuals with ID. This pilot study will show how the use of a technological tool, such as mHealth, can be tested in research involving individuals with ID. The use of technology as a means of improving health has been explored to some extent in this population [20,21,55-57], but limited studies have investigated the use of mHealth for improving levels of PA for individuals with ID [25,58].

The quantitative measurements at baseline and follow-up may reveal differences in PA levels, self-efficacy of PA or in other psychosocial variables. In addition, we expect to find goal achievement among the participants by follow-up. We do not expect to detect any effects of the trial as it is a pilot study [59]. In addition, we expect a possible finding to be that the use of 2 activity trackers, Fitbit and Axivity, will strengthen the quality of activity data. We will gain experience in using one commercial and one research-friendly activity tracker. This experience can be useful in future trials.

In this pilot study, we expect that the use of the mHealth tool will rely on the caregivers or staffs’ involvement. The study will, therefore, offer insight into the inclusion of support persons in the planning of PA using mHealth tools, as previous research has shown a greater motivation for PA in individuals with ID if support persons are engaged and included in the activity [26]. It will also explore how the use of goal attainment for adults with ID can be used in a research project on PA promotion [60]. The influence of the use of technology on motivation for PA is being investigated through qualitative methods.

### Potential Limitations and Implications

This study will have several limitations. The sample size will be small, which will influence the representativeness of the quantitative data. For the qualitative data, a sample of 10 participants is expected to be of good quality if data saturation is achieved. Another limitation in the data collection can be that having 2 activity trackers might not be accepted by participants, resulting in no measurement if the participants stop using them. Participants or caretakers who agree to be included in the pilot study might be interested or engaged in PA promotion and the variation in the included participants regarding interest in PA might be scarce.

The study has potentially important implications for both individuals with ID and their support networks. The study will be presented at conferences and published in recognized international peer-reviewed journals.

### Conclusions

This pilot study will evaluate the feasibility and acceptability of study procedures of the intervention arm of a planned RCT. This can, in turn, address feasibility issues, improve study procedures, and estimate effectiveness of the study measures. We will investigate how technology can be used to influence motivation for PA, which can help guide and improve future PA interventions involving the use of technology. Investigating new ways of enhancing PA for individuals with ID is important to ensure better health and quality of life for this group.

## Appendix

Multimedia Appendix 1 Screenshots of the interface options of the Active Leisure app: symbols only, easy to read text, or plain text. The app also has read-aloud capabilities.

Multimedia Appendix 2 Screenshots of the augmented reality application “Sorterius”.

## Abbreviations

CONSORT: Consolidated Standards of Reporting Trials
ID: intellectual disability
mHealth: mobile health
NOHID: Norwegian Health in Intellectual Disability
PA: physical activity
RCT: randomized controlled trial
REDCap: Research Electronic Data Capture

## Data Availability

In this study, participants will not be asked to approve data sharing in the informed consent because some data will include sensitive information, and the small sample makes it possible to identify participants even if the data are deidentified. Moreover, sharing of qualitative data can be difficult. In the ethics committee approval, sharing of data has not been accepted. In future trials, an ethics approval application may include requests for data sharing.
