# Supplementary figures and images for: mHealth Support to Stimulate Physical Activity in Individuals With Intellectual Disability: Protocol for a Mixed Methods Pilot Study
Source: JMIR Res Protoc. 2022 Sep 15;11(9):e37849. doi: 10.2196/37849 (PMC9523523; doi:10.2196/37849)

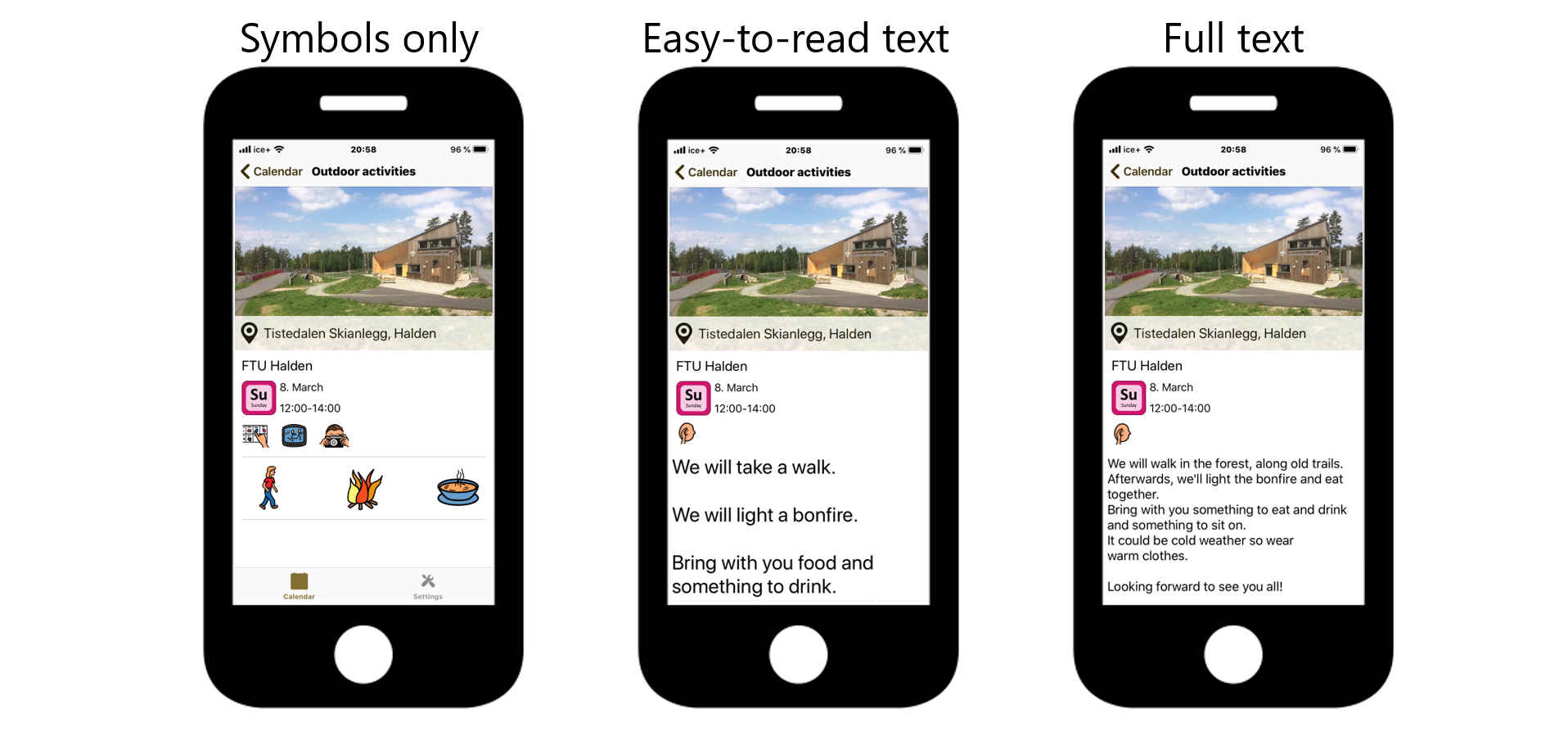

Supplement: Multimedia Appendix 1 [file resprot_v11i9e37849_app1.png]

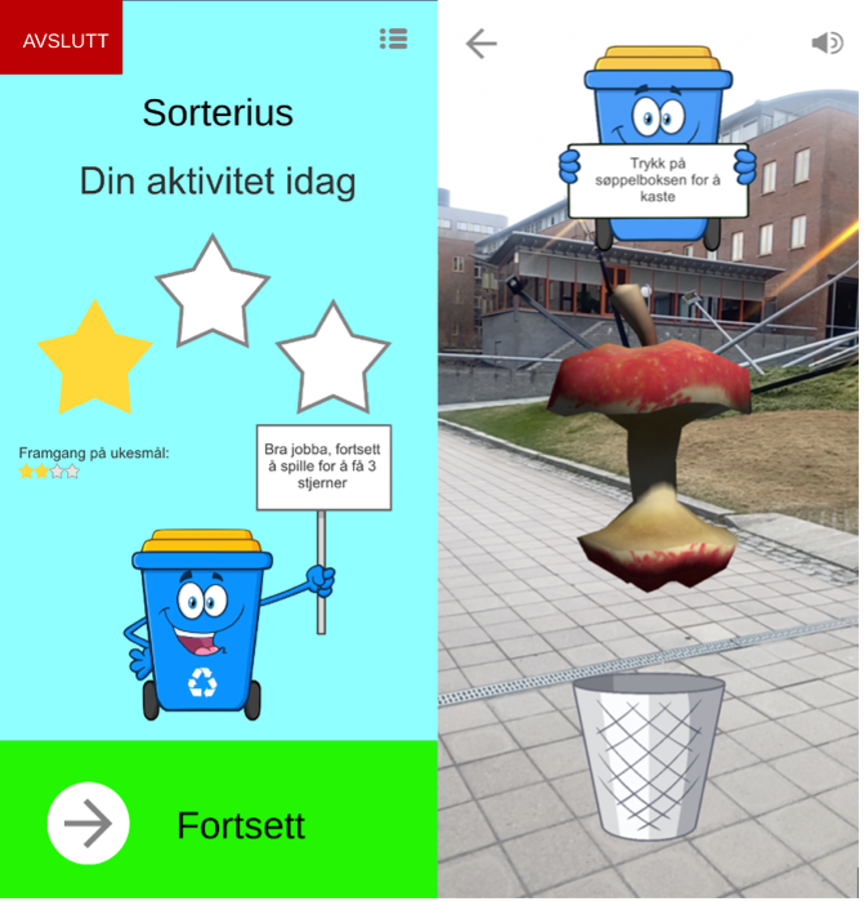

Supplement: Multimedia Appendix 2 [file resprot_v11i9e37849_app2.png]
